# Influenza and Antibody-Dependent Cellular Cytotoxicity

**DOI:** 10.3389/fimmu.2019.01457

**Published:** 2019-06-25

**Authors:** Tarra A. Von Holle, M. Anthony Moody

**Affiliations:** ^1^Duke University Human Vaccine Institute, Duke University School of Medicine, Durham, NC, United States; ^2^Department of Immunology, Duke University School of Medicine, Durham, NC, United States; ^3^Department of Pediatrics, Duke University School of Medicine, Durham, NC, United States

**Keywords:** ADCC—antibody dependent cellular cytotoxicity, influenza, antibodies, animal models, vaccine targets, mAb

## Abstract

Despite the availability of yearly vaccinations, influenza continues to cause seasonal, and pandemic rises in illness and death. An error prone replication mechanism results in antigenic drift and viral escape from immune pressure, and recombination results in antigenic shift that can rapidly move through populations that lack immunity to newly emergent strains. The development of a “universal” vaccine is a high priority and many strategies have been proposed, but our current understanding of influenza immunity is incomplete making the development of better influenza vaccines challenging. Influenza immunity has traditionally been measured by neutralization of virions and hemagglutination inhibition, but in recent years there has been a growing appreciation of other responses that can contribute to protection such as antibody-dependent cellular cytotoxicity (ADCC) that can kill influenza-infected cells. ADCC has been shown to provide cross-strain protection and to assist in viral clearance, making it an attractive target for “universal” vaccine designs. Here we provide a brief overview of the current state of influenza research that leverages “the other end of the antibody.”

## Introduction

Influenza causes 3–5 million cases of severe illness and 290,000–650,000 deaths annually (1). Influenza is caused by orthomyxoviruses that have a segmented, negative-strand RNA genome that encodes its own RNA-dependent RNA polymerase that results in ~1 error per replicated genome (2–4). Accumulating errors cause small changes over time that allow viral escape (5, 6), a process called antigenic drift. Reassorting of the segmented influenza genome can produce novel influenza strains to which there is no preexisting immunity in the human population, a process called antigenic shift, and it is thought this mechanism produced the 1918, 1957, and 1968 pandemics (7).

The primary means of combatting both seasonal and pandemic influenza are quarantine and isolation (8), strict hygiene (9), and vaccination (10–12). Influenza vaccines were developed starting in the 1940s (13–16), and seasonal vaccines now include multiple antigens either as inactivated or live-attenuated products (17–20). Selection of representative influenza strains requires the ongoing worldwide analysis of circulating influenza (21), leading to potential mismatch with low vaccine efficacy (22). Public health leaders have called for the development of “universal” influenza vaccines (23), but at this time it is not clear what kinds of immunity such a vaccine should elicit.

**Figure 1:**
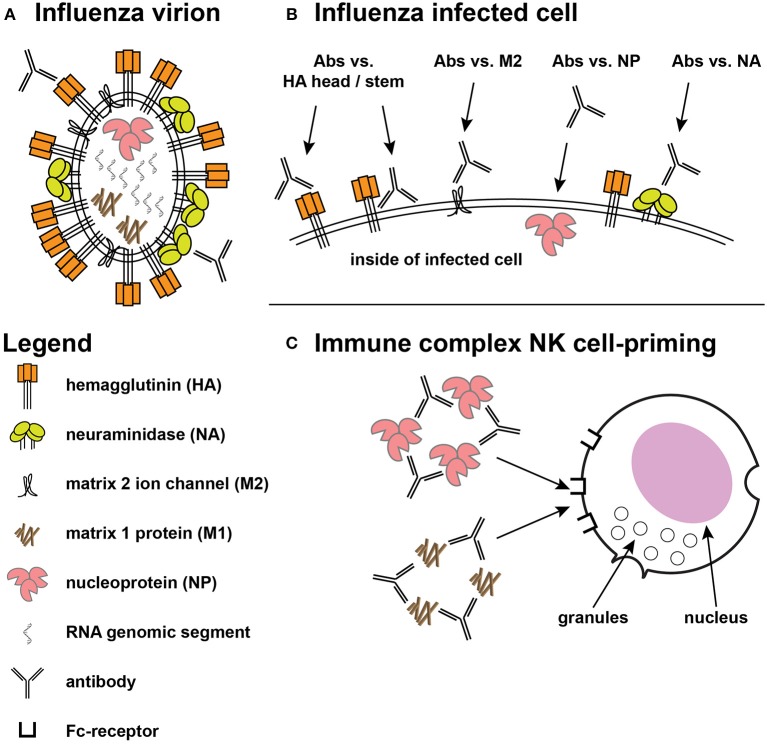
Targets for protective influenza responses. **(A)** Influenza virion showing the tight packing of viral hemagglutinin (HA), neuraminidase (NA), and matrix 2 (M2) ion channels on the surface. Inside the virion are the 8 genomic segments, nucleoprotein (NP), and the structural matrix 1 (M1) protein. **(B)** The surface of an infected cell shows the same antigens spread out on the surface, providing access for ADCC-mediating antibodies directed at the HA head and stem, along with greater access to the M2 ion channel. Antibodies against NP have also shown ADCC activity. **(C)** Immune complexes of NP and M1 have been shown to prime NK cells to secrete cytokines.

Vaccines can prevent symptomatic disease, reduce disease duration, and reduce viral shedding, and infectivity to other persons. To accomplish these, a vaccine can elicit responses that inhibit influenza virions and/or enhance the clearance of influenza-infected cells. The influenza virion has three virally-encoded surface antigens (Figure 1A): a trimeric glycoprotein hemagglutinin (HA) that binds to sialic acid on cell surface receptors promoting virion endocytosis followed by fusion of viral and host cell membranes, a tetrameric neuraminidase (NA) that cleaves sialic acid to release virions from infected cells, and enhance passage through respiratory mucins, and a proton channel (M2 matrix) that helps the release of the viral genome after acidification in endocytic vacuoles. These proteins are present in most split-virus vaccine products (24), but the exact contribution of each of these, or of other influenza proteins that are not surface expressed, to vaccine efficacy is unclear.

## ADCC Activity Against Influenza

Antibody specificity is mediated by binding of Fab (fragment antigen binding) domains to their antigenic target, while the other end of the antibody is a constant region, the Fc (fragment crystallizable) domain, that provides a link between antibody recognition of infected cells and effector cells. NK cells, monocytes/macrophages, and neutrophils all have Fc-receptors (FcRs) on their surface, and the combination of both activating and inhibitory signals direct the immune response (25). In humans, NK cells are generally thought of as the primary effector for ADCC, but ADCC of influenza-infected cells has been demonstrated using neutrophils, monocytes, lymphocytes, and cord blood cells (26–28).

An increasing body of literature has demonstrated the possible protective role of non-neutralizing antibodies for pathogens as diverse as HIV-1 (29, 30), herpes simplex virus (31–33), Ebola (34, 35), and influenza (36–38). Influenza-infected cells can be recognized by antibodies that bind to proteins that play important roles within the viral life cycle. As noted above, HA initiates the viral life cycle by binding sialic acid and mediating viral entry into cells via fusion after receptor-mediated endocytosis (2). During this process, M2 matrix channels promote pH equilibration, a critical step in the release of the viral genome from the endosome (39). After influenza genome replication and protein synthesis, viral components are transferred to the plasma membrane where viral budding occurs. NA removes sialic acid from glycoproteins allowing the release of newly assembled virions from the surface of infected cells and preventing aggregation (2). HA has been the focus of most vaccine designs because HA evolves more rapidly than other antigens (40), but more conserved antigens like NA, M2, and nucleoprotein (NP) have also been considered attractive targets for vaccine designs. Antibodies against these other antigens do not appear to directly prevent infection but can target infected cells (19), and antibodies against all of these proteins have mediated killing of infected cells *in vitro* (Figure 1B).

Antibody-dependent cellular cytotoxicity (ADCC) against influenza was described by Greenberg et al. (26) and ADCC activity can be mediated by multiple cell types (27, 28, 41) and arises after infection or vaccination (41–43). Most early work suggested that ADCC was primarily directed against HA (44), although the contribution of other antigens to ADCC could not be ruled out (43). Study of influenza-specific monoclonal antibodies (mAbs) have shown that antibodies directed against the globular head often lack breadth while those directed against the more conserved HA stem can recognize multiple strains and subtypes of HA (4). Both specificities of HA antibodies have mediated ADCC (Figure 1B) but antibodies with neutralization and hemagglutination inhibition (HAI) activity tend to be directed against the HA head domain (Figure 1A).

ADCC-mediating antibodies also target other proteins on the virus surface (Figure 1B). Murine mAbs against influenza B NA mediated ADCC and provided protection in mouse challenge (45), but whether this activity contributes significantly to human protection is not known. NA antibody titers have correlated with protection against influenza infection in humans (46–50), but NA responses have not been studied at the same level of molecular detail as HA responses (51). NA antibodies are thought to act by decorating infected cells thus making them targets for complement fixation and ADCC, and by inhibiting enzymatic activity and preventing virions from escaping respiratory mucins (19). NA is present in most vaccines derived from influenza virions (24), but few vaccine candidates focus on NA antigens and their relative contribution to protection is not well-quantified. There is growing interest in NA as a component vaccine-mediated protection, and the recent formation of the NAction! working group (51) is focused on filling key knowledge gaps.

M2 is a highly conserved antigen necessary for the virus life cycle (39) and a number of studies showed antibodies to M2 mediated ADCC and protected in mouse studies (36, 52, 53). M2 is present at low copy number on virions and may be occluded by the larger HA and NA proteins (19) (Figure 1A), and while vaccine candidates based on M2 have advanced to human clinical trials (54, 55), it is not yet known whether these approaches will provide broad cross-protection when tested in human efficacy trials.

Curiously, a protein that might not be expected on the surface of either virions or virus-infected cells has been shown to mediate ADCC *in vitro*. Influenza nucleoprotein (NP) is involved in replication and packaging of virus RNA segments into virions (56), but studies have shown that NP is surface expressed on infected cells (57, 58). Humans given seasonal vaccine demonstrated H7N9 cross-reactive ADCC activity that correlated with binding to NP (59). Antibodies against both NP and the internal RNA-binding and structural matrix 1 (M1) protein did not mediate ADCC against target cells expressing those specific antigens, but immune complexes of those proteins primed natural killer (NK) cells to secrete cytokines (60) (Figure 1C); the authors suggested such activity could contribute to an anti-viral environment. Whether these kinds of activities play an important role in cross-protection against human infection is not yet known.

## ADCC in Mouse Models

The receptors and cell types important for human immune response may not have the same effect or use the same pathways in animal models. In fact, there are numerous differences among antibody isotypes and subclasses (61) in animal models typically used for immune studies, as well as in FcR sequences and cellular distributions (62), and such differences may result in species-specific mechanisms of ADCC activity against influenza.

With that caveat, mouse models have provided suggestive evidence for the kinds of immunity that might be protective in humans. DiLillo et al. administered human mAbs to transgenic mice that expressed human FcRs, and demonstrated that for both HA stem-directed antibodies (37) and HA head-directed antibodies (63) that Fc-FcR interactions were required for protection against lethal challenge. Interestingly, transgenic mice receiving neutralizing anti-HA and anti-NA antibodies were only protected against challenge if they were matched for Fc-Fc?R binding while strain specific anti-HA and anti-NA antibodies protected regardless of Fc-FcR matching (63). Similar studies of antibodies against M2 have also shown Fc-FcR dependence, with IgG1 M2-directed antibodies requiring matching FcRs to protect against lethal challenge (52, 53).

Wild-type mice have also demonstrated ADCC-mediating antibody protection. For example, murine antibodies generated against H7N9 influenza were protective, but ADCC-mediating antibodies required efficient Fc-FcR interaction to protect (64). Vaccination against H5N1 that elicited ADCC activity protected against lethal H5N8 challenge in mice (65), and protection against H7 influenza challenge also correlated with ADCC activity (66). Passive transfer of HA stem-specific human sera demonstrated protection against lethal challenge in a manner highly correlated with ADCC activity (67). Protection is not limited to HA-directed antibodies, as antibodies against influenza B NA also mediate ADCC and protect against lethal virus challenge (45).

However, not all mouse studies have suggested a protective effect of ADCC. Evaluation of candidate vaccines that targeted specific epitopes on the HA head domain were capable of eliciting potent ADCC responses *in vitro*, but mice given these immunogens were more sensitive to lethal challenge (68, 69), and examination of lung tissue suggested damage caused by the immune response. Whether this kind of damage could occur in humans is not known.

## Other Non-Primate Models

Swine are susceptible to influenza and they are thought to be a key species in development of new strains by antigenic shift (70). Because of the major economic impact of swine influenza disease, vaccination is common and new vaccine candidates are tested in swine (71). Experimental infection models have been used to test swine for protection against challenge following vaccination (72) and as a model of enhanced influenza disease (73). Studies in this latter model have suggested that a lack of neutralizing activity against the challenge strain combined with ADCC-mediating antibodies can produce enhanced disease in swine (73, 74), suggesting that caution may be appropriate in the development of vaccines that do not elicit traditional correlates of influenza protection (75). Furthermore, differences between swine and human FcRs make direct measures of ADCC in swine challenging, and have confounded passive infusion studies of human antibodies (76). The swine model is important for improving our understanding of influenza pathogenesis, but it remains to be seen whether success or failure in the swine model will directly translate to human trials.

The ferret model may also provide insights into influenza immunity, although direct measures of ADCC activity in ferrets is also challenging. For example, immunization of ferrets to elicit HA stem-directed antibodies showed that ADCC in a reporter assay correlated with protection, though the assay used human FcRs due to the lack of ferret-specific reagents (77). Passive transfer of immune sera directed against the HA stem (78), infusion of ADCC-mediating mAbs (79), and immunization to induce ADCC-mediating antibodies (65) have all protected ferrets against heterologous influenza challenges. Unfortunately, the lack of ferret-specific reagents limits the depth of investigation possible at this time, and as with swine, it is not clear whether these studies will translate directly to human trials.

## Non-human Primate Studies

Non-human primate models have not traditionally been used for influenza research due to high cost and the low level of symptoms following experimental infection (80), but recently a number of studies have evaluated the protective capacity of ADCC in non-human primates. For example, protection was observed in rhesus macaques infected with a pre-pandemic H1N1 A/Kawasaki/173/2001 who were subsequently challenged with H1N1 A/California/04/2009; in this study ADCC activity correlated with control of the second H1N1 infection (81). Protection was also observed in a study of H5 immunization of rhesus macaques challenged with pandemic H1N1, and ADCC activity correlated with reduced viral shedding after infection (82). In cynomolgus macaques, vaccines that elicit ADCC-mediating antibodies have been shown to decrease shedding of influenza (83), and passive infusion of a human ADCC-mediating mAb protected against infection (84). However, given the differences between human and non-human primate Fc-FcR biology (85), it is not clear if non-human primate studies will be predictive of human study outcomes.

## Human Studies

Human studies have focused on examining people after natural infection, vaccination, and/or the isolation of human monoclonal antibodies. Natural infection studies have shown that influenza can imprint the immune system in a manner that provides protection against heterologous influenza exposure. For example, healthy adults and children in the US were found to have ADCC-mediating antibodies against H5N1 and H7N9 strains (86), despite the fact that those strains have not circulated in the US. Intravenous immune globulin, a product derived from plasma donation, contained ADCC-mediating antibodies that cross-reacted with multiple influenza strains; e.g., immune globulin collected from donors prior to the emergence of the 2009 pandemic strain was active against the newly emergent strain (87). During the last influenza pandemic a number of studies suggested that older adults were less likely to be infected by the newly emergent strain, and older persons were more likely to have ADCC-mediating antibodies against the 2009 H1N1 pandemic strain (88), suggesting that these responses may have contributed to the relative protection observed. However, such antibodies were not well correlated with protection in children (89, 90), indicating that *in vitro* activity alone may not provide an accurate correlate of efficacy.

ADCC-mediating antibodies that arise during severe infection may also correlate with outcomes. A study of seasonal and H7N9 human infections demonstrated that persons who did not survive infection were more likely to have low ADCC activity (91). It is not known whether ADCC-mediating antibodies being present prior to infection would have been protective.

Vaccine studies have examined the ability of different constructs to elicit cross-reactive ADCC-mediating antibodies. For example, seasonal vaccination of older adults indicated that they had strong boosting of ADCC activity, including activity against H5 and H7 strains (92). Similarly, a study of H7 vaccination found ADCC activity against many group 2 influenza strains (93), and H5 vaccination induced ADCC responses to multiple strains (94).

Testing of many of human-derived antibodies showed protection in mouse studies as described above and have helped define vaccine design targets. For example, antibodies against the vestigial esterase domain of H3 HA (95) or influenza B (96) mediate ADCC and block other viral activities, but whether such antibodies can be easily elicited by vaccination and protect is not known. Others have examined why HA head-directed antibodies are less efficient at mediating ADCC (97) or why antibodies that bind at or near the sialic acid receptor binding site of HA lack ADCC activity (98, 99). Data suggested that ADCC activity against influenza-infected cells requires that sialic acid on NK cells must also bind HA. Several studies have identified possible vaccine targets on HA, including a pH-sensitive epitope on H7 HA (100), receptor binding site antibodies for influenza B (79), and the influenza B HA stem (101). Furthermore, recently isolated human antibodies against epitopes in the HA trimer interface provided protection in an Fc-dependent manner (38, 102), and such antibodies were elicited in animal models by vaccination (103). These studies suggest that probing the human immune system could identify additional targets for vaccine design.

Most human studies measure responses to influenza infection or vaccination, and some correlate those responses with epidemiologic data, but far fewer perform experimental infectious challenge to correlate a response with protection. In 2016, Jegaskanda et al. reported that high levels of pre-existing ADCC-mediating antibodies protected against experimental infection (104), suggesting that if ADCC activity is present prior to infection it can protect. However, it is not clear whether ADCC activity alone is protective, and as of this writing, there do not appear to be any reports of human challenge studies investigating the protective capacity of passively administered ADCC-mediating influenza antibodies. Essentially all humans over the age of 6 years have evidence of prior influenza infection (105), meaning that vaccination or passive infusion studies of ADCC-based protection in humans have to be interpreted in the light of prior immunity. Despite this, the studies described in this review and other work are leading to new vaccine designs, and it is hoped that one or more of these new designs will provide long-lasting protection to all people worldwide.

## Conclusions

Influenza remains an important cause of human disease that often resists effective control by current vaccination strategies and there is a current push for the development of “universal vaccine” candidates. Antibody binding to Fc receptors with effector cell activation has been shown to protect in numerous animal models and harnessing this activity could be an important component of universal vaccine designs. ADCC and other activities based on the “other end of the antibody,” in combination with traditional activities like neutralization, might be harnessed to contribute to protection from this ever-changing pathogen.

## Author Contributions

TV wrote the first draft and participated in editing. MM edited the draft and prepared the final version.

### Conflict of Interest Statement

The authors declare that the research was conducted in the absence of any commercial or financial relationships that could be construed as a potential conflict of interest.

## References

[1] World Health Organization Influenza Update – 331. (2018). Available online at: https://www.who.int/influenza/surveillance_monitoring/updates/latest_update_GIP_surveillance/en/ (accessed Jan 01, 2019).

[2] Bouvier NM Palese P. The biology of influenza viruses. Vaccine. (2008) 26 (Suppl. 4):D49–53. 10.1016/j.vaccine.2008.07.039 19230160 PMC3074182

[3] Boivin S Cusack S Ruigrok RW Hart DJ. Influenza A virus polymerase: structural insights into replication and host adaptation mechanisms. J Biol Chem. (2010) 285:28411–7. 10.1074/jbc.R110.117531 20538599 PMC2937865

[4] Air GM. Influenza virus antigenicity and broadly neutralizing epitopes. Curr Opin Virol. (2015) 11:113–21. 10.1016/j.coviro.2015.03.006 25846699 PMC4456283

[5] Huang KY Rijal P Schimanski L Powell TJ Lin TY McCauley JW. Focused antibody response to influenza linked to antigenic drift. J Clin Invest. (2015) 125:2631–45. 10.1172/JCI81104 26011643 PMC4613558

[6] Schmidt AG Do KT McCarthy KR Kepler TB Liao HX Moody MA. Immunogenic stimulus for germline precursors of antibodies that engage the influenza hemagglutinin receptor-binding site. Cell Rep. (2015) 13:2842–50. 10.1016/j.celrep.2015.11.063 26711348 PMC4698027

[7] Kilbourne ED. Influenza pandemics of the 20th century. Emerg Infect Dis. (2006) 12:9–14. 10.3201/eid1201.051254 16494710 PMC3291411

[8] World Health Organization Writing G Bell D Nicoll A Fukuda K Horby P Monto A. Non-pharmaceutical interventions for pandemic influenza, national and community measures. Emerg Infect Dis. (2006) 12:88–94. 10.3201/eid1201.051371 16494723 PMC3291415

[9] Blanco N Eisenberg MC Stillwell T Foxman B. What transmission precautions best control influenza spread in a hospital? Am J Epidemiol. (2016) 183:1045–54. 10.1093/aje/kwv293 27188950

[10] Grohskopf LA Sokolow LZ Broder KR Walter EB Bresee JS Fry AM. Prevention and control of seasonal influenza with vaccines: recommendations of the advisory committee on immunization practices - United States, 2017-18 influenza season. MMWR Recomm Rep. (2017) 66:1–20. 10.15585/mmwr.rr6602a1 28841201 PMC5837399

[11] Grohskopf LA Sokolow LZ Fry AM Walter EB Jernigan DB. Update: ACIP recommendations for the use of quadrivalent live attenuated influenza vaccine (LAIV4) - United States, 2018-19 influenza season. MMWR Morb Mortal Wkly Rep. (2018) 67:643–5. 10.15585/mmwr.mm6722a5 29879095 PMC5991811

[12] Robinson CL Romero JR Kempe A Pellegrini C Szilagyi P. Advisory committee on immunization practices recommended immunization schedule for children and adolescents aged 18 years or younger - United States, 2018. MMWR Morb Mortal Wkly Rep. (2018) 67:156–7. 10.15585/mmwr.mm6705e2 29420458 PMC5812475

[13] Francis T Salk JE Pearson HE Brown PN Protective effect of vaccination against induced influenza A. J Clin Invest. (1945) 24:536–46. 10.1172/JCI101633 16695243 PMC435485

[14] Salk JE Pearson HE Brown PN Francis T. Protective effect of vaccination against induced influenza B. J Clin Invest. (1945) 24:547–53. 10.1172/JCI101634 16695244 PMC435486

[15] Francis T Salk JE Quilligan JJ. Experience with vaccination against influenza in the spring of 1947: a preliminary report. Am J Public Health Nations Health. (1947) 37:1013–6. 10.2105/AJPH.37.8.1013 18016577 PMC1623895

[16] Salk JE Suriano PC. Importance of antigenic composition of influenza virus vaccine in protecting against the natural disease; observations during the winter of 1947-1948. Am J Public Health Nations Health. (1949) 39:345–55. 10.2105/AJPH.39.3.345 18124075 PMC1527846

[17] Allison JE Glezen WP Taber LH Paredes A Webster RG. Reactogenicity and immunogenicity of bivalent influenza A and monovalent influenza B virus vaccines in high-risk children. J Infect Dis. (1977) 136 (Suppl):S672–6. 10.1093/infdis/136.Supplement_3.S672 606790

[18] Belshe RB Gruber WC Mendelman PM Mehta HB Mahmood K Reisinger K. Correlates of immune protection induced by live, attenuated, cold-adapted, trivalent, intranasal influenza virus vaccine. J Infect Dis. (2000) 181:1133–7. 10.1086/315323 10720541

[19] Krammer F Palese P. Advances in the development of influenza virus vaccines. Nat Rev Drug Discov. (2015) 14:167–82. 10.1038/nrd4529 25722244

[20] Soema PC Kompier R Amorij JP Kersten GF. Current and next generation influenza vaccines: formulation and production strategies. Eur J Pharm Biopharm. (2015) 94:251–63. 10.1016/j.ejpb.2015.05.023 26047796

[21] Barr IG McCauley J Cox N Daniels R Engelhardt OG Fukuda K. Epidemiological, antigenic and genetic characteristics of seasonal influenza A(H1N1), A(H3N2) and B influenza viruses: basis for the WHO recommendation on the composition of influenza vaccines for use in the 2009–2010 northern hemisphere season. Vaccine. (2010) 28:1156–67. 10.1016/j.vaccine.2009.11.043 20004635

[22] Tricco AC Chit A Soobiah C Hallett D Meier G Chen MH. Comparing influenza vaccine efficacy against mismatched and matched strains: a systematic review and meta-analysis. BMC Med. (2013) 11:153. 10.1186/1741-7015-11-153 23800265 PMC3706345

[23] Erbelding EJ Post D Stemmy E Roberts PC Augustine AD Ferguson S. A universal influenza vaccine: the strategic plan for the national institute of allergy and infectious diseases. J Infect Dis. (2018) 218:347–54. 10.1093/infdis/jiy103 29506129 PMC6279170

[24] Renfrey S Watts A. Morphological and biochemical characterization of influenza vaccines commercially available in the United Kingdom. Vaccine. (1994) 12:747–52. 10.1016/0264-410X(94)90227-5 8091854

[25] Nimmerjahn F Ravetch JV. Fcgamma receptors as regulators of immune responses. Nat Rev Immunol. (2008) 8:34–47. 10.1038/nri2206 18064051

[26] Greenberg SB Criswell BS Six HR Couch RB. Lymphocyte cytotoxicity to influenza virus-infected cells. II requirement for antibody and non-T lymphocytes. J Immunol. (1977) 119:2100–6. 334984

[27] Hashimoto G Wright PF Karzon DT. Antibody-dependent cell-mediated cytotoxicity against influenza virus-infected cells. J Infect Dis. (1983) 148:785–94. 10.1093/infdis/148.5.785 6605395

[28] Hashimoto G Wright PF Karzon DT. Ability of human cord blood lymphocytes to mediate antibody-dependent cellular cytotoxicity against influenza virus-infected cells. Infect Immun. (1983) 42:214–8. 6604697 10.1128/iai.42.1.214-218.1983 PMC264545

[29] Forthal DN Landucci G Daar ES. Antibody from patients with acute human immunodeficiency virus (HIV) infection inhibits primary strains of HIV type 1 in the presence of natural-killer effector cells. J Virol. (2001) 75:6953–61. 10.1128/JVI.75.15.6953-6961.2001 11435575 PMC114423

[30] Haynes BF Gilbert PB McElrath MJ Zolla-Pazner S Tomaras GD Alam SM. Immune-correlates analysis of an HIV-1 vaccine efficacy trial. N Engl J Med. (2012) 366:1275–86. 10.1056/NEJMoa1113425 22475592 PMC3371689

[31] Kohl S Loo LS Pickering LK Protection of neonatal mice against herpes simplex viral infection by human antibody and leukocytes from adult, but not neonatal humans. J Immunol. (1981) 127:1273–5. 7276559

[32] Kohl S Loo LS. Protection of neonatal mice against herpes simplex virus infection: probable *in vivo* antibody-dependent cellular cytotoxicity. J Immunol. (1982) 129:370–6. 6282968

[33] Wang K Tomaras GD Jegaskanda S Moody MA Liao HX Goodman KN. Monoclonal antibodies, derived from humans vaccinated with the RV144 HIV vaccine containing the HVEM binding domain of herpes simplex virus (HSV) glycoprotein D, neutralize HSV infection, mediate antibody-dependent cellular cytotoxicity, and protect mice from ocular challenge with HSV-1. J Virol. (2017) 91. 10.1128/JVI.00411-17 28701403 PMC5599770

[34] Gunn BM Yu WH Karim MM Brannan JM Herbert AS Wec AZ. A role for Fc function in therapeutic monoclonal antibody-mediated protection against ebola virus. Cell Host Microbe. (2018) 24:221–33 e225. 10.1016/j.chom.2018.07.009 30092199 PMC6298217

[35] Saphire EO Schendel SL Fusco ML Gangavarapu K Gunn BM Wec AZ. Systematic analysis of monoclonal antibodies against ebola virus GP defines features that contribute to protection. Cell. (2018) 174:938–52.e913. 10.1016/j.cell.2018.07.033 30096313 PMC6102396

[36] Jegerlehner A Schmitz N Storni T Bachmann MF. Influenza A vaccine based on the extracellular domain of M2: weak protection mediated via antibody-dependent NK cell activity. J Immunol. (2004) 172:5598–605. 10.4049/jimmunol.172.9.5598 15100303

[37] DiLillo DJ Tan GS Palese P Ravetch JV. Broadly neutralizing hemagglutinin stalk-specific antibodies require FcgammaR interactions for protection against influenza virus *in vivo*. Nat Med. (2014) 20:143–51. 10.1038/nm.3443 24412922 PMC3966466

[38] Watanabe A McCarthy KR Kuraoka M Schmidt AG Adachi Y Onodera T. Antibodies to a conserved influenza head interface epitope protect by an IgG subtype-dependent mechanism. Cell. (2019) 177:1124–35 e1116. 10.1016/j.cell.2019.03.048 31100267 PMC6825805

[39] Pielak RM Chou JJ. Influenza M2 proton channels. Biochim Biophys Acta. (2011) 1808:522–9. 10.1016/j.bbamem.2010.04.015 20451491 PMC3108042

[40] Kilbourne ED Johansson BE Grajower B. Independent and disparate evolution in nature of influenza A virus hemagglutinin and neuraminidase glycoproteins. Proc Natl Acad Sci USA. (1990) 87:786–90. 10.1073/pnas.87.2.786 2300562 PMC53351

[41] Greenberg SB Criswell BS Six HR Couch RB. Lymphocyte cytotoxicity to influenza virus-infected cells: response to vaccination and virus infection. Infect Immun. (1978) 20:640–5. 669816 10.1128/iai.20.3.640-645.1978 PMC421906

[42] Greenberg SB Six HR Drake S Couch RB. Cell cytotoxicity due to specific influenza antibody production *in vitro* after recent influenza antigen stimulation. Proc Natl Acad Sci USA. (1979) 76:4622–6. 10.1073/pnas.76.9.4622 291990 PMC411631

[43] Vella S Rocchi G Resta S Marcelli M De Felici A. Antibody reactive in antibody-dependent cell-mediated cytotoxicity following influenza virus vaccination. J Med Virol. (1980) 6:203–11. 10.1002/jmv.1890060303 7229627

[44] Jegaskanda S Reading PC Kent SJ. Influenza-specific antibody-dependent cellular cytotoxicity: toward a universal influenza vaccine. J Immunol. (2014) 193:469–75. 10.4049/jimmunol.1400432 24994909

[45] Wohlbold TJ Podolsky KA Chromikova V Kirkpatrick E Falconieri V Meade P. Broadly protective murine monoclonal antibodies against influenza B virus target highly conserved neuraminidase epitopes. Nat Microbiol. (2017) 2:1415–24. 10.1038/s41564-017-0011-8 28827718 PMC5819343

[46] Murphy BR Kasel JA Chanock RM. Association of serum anti-neuraminidase antibody with resistance to influenza in man. N Engl J Med. (1972) 286:1329–32. 10.1056/NEJM197206222862502 5027388

[47] Couch RB Kasel JA Gerin JL Schulman JL Kilbourne ED. Induction of partial immunity to influenza by a neuraminidase-specific vaccine. J Infect Dis. (1974) 129:411–20. 10.1093/infdis/129.4.411 4593871

[48] Couch RB Atmar RL Franco LM Quarles JM Wells J Arden N. Antibody correlates and predictors of immunity to naturally occurring influenza in humans and the importance of antibody to the neuraminidase. J Infect Dis. (2013) 207:974–81. 10.1093/infdis/jis935 23307936 PMC3633450

[49] Monto AS Petrie JG Cross RT Johnson E Liu M Zhong W. Antibody to influenza virus neuraminidase: an independent correlate of protection. J Infect Dis. (2015) 212:1191–9. 10.1093/infdis/jiv195 25858957

[50] Memoli MJ Shaw PA Han A Czajkowski L Reed S Athota R. Evaluation of antihemagglutinin and antineuraminidase antibodies as correlates of protection in an influenza A/H1N1 virus healthy human challenge model. MBio. (2016) 7:e00417–6. 10.1128/mBio.00417-16 27094330 PMC4959521

[51] Krammer F Fouchier RAM Eichelberger MC Webby RJ Shaw-Saliba K Wan H. NAction! How can neuraminidase-based immunity contribute to better influenza virus vaccines? MBio. (2018) 9:e02332–17. 10.1128/mBio.02332-17 29615508 PMC5885027

[52] El Bakkouri K Descamps F De Filette M Smet A Festjens E Birkett A. Universal vaccine based on ectodomain of matrix protein 2 of influenza A: Fc receptors and alveolar macrophages mediate protection. J Immunol. (2011) 186:1022–31. 10.4049/jimmunol.0902147 21169548

[53] Van den Hoecke S Ehrhardt K Kolpe A El Bakkouri K Deng L Grootaert H. Hierarchical and redundant roles of activating fcgammars in protection against influenza disease by M2e-specific IgG1 and IgG2a antibodies. J Virol. (2017) 91:e02500–16. 10.1128/JVI.02500-16 28077656 PMC5355615

[54] Turley CB Rupp RE Johnson C Taylor DN Wolfson J Tussey L. Safety and immunogenicity of a recombinant M2e-flagellin influenza vaccine (STF2.4xM2e) in healthy adults. Vaccine. (2011) 29:5145–52. 10.1016/j.vaccine.2011.05.041 21624416

[55] Ibanez LI Roose K De Filette M Schotsaert M De Sloovere J Roels S. M2e-displaying virus-like particles with associated RNA promote T helper 1 type adaptive immunity against influenza A. PLoS ONE. (2013) 8:e59081. 10.1371/journal.pone.0059081 23527091 PMC3601086

[56] Portela A Digard P. The influenza virus nucleoprotein: a multifunctional RNA-binding protein pivotal to virus replication. J Gen Virol. (2002) 83(Pt 4):723–34. 10.1099/0022-1317-83-4-723 11907320

[57] Yewdell JW Frank E Gerhard W. Expression of influenza A virus internal antigens on the surface of infected P815 cells. J Immunol. (1981) 126:1814–9. 7217668

[58] Fujimoto Y Tomioka Y Takakuwa H Uechi G Yabuta T Ozaki K. Cross-protective potential of anti-nucleoprotein human monoclonal antibodies against lethal influenza A virus infection. J Gen Virol. (2016) 97:2104–16. 10.1099/jgv.0.000518 27260213

[59] Jegaskanda S Co MDT Cruz J Subbarao K Ennis FA Terajima M. Induction of H7N9-cross-reactive antibody-dependent cellular cytotoxicity antibodies by human seasonal influenza A viruses that are directed toward the nucleoprotein. J Infect Dis. (2017) 215:818–23. 10.1093/infdis/jiw629 28011910 PMC5853654

[60] Vanderven HA Ana-Sosa-Batiz F Jegaskanda S Rockman S Laurie K Barr I. What lies beneath: antibody dependent natural killer cell activation by antibodies to internal influenza virus proteins. EBioMed. (2016) 8:277–90. 10.1016/j.ebiom.2016.04.029 27428437 PMC4919476

[61] Stavnezer J Amemiya CT. Evolution of isotype switching. Semin Immunol. (2004) 16:257–75. 10.1016/j.smim.2004.08.005 15522624

[62] Kacskovics I. Fc receptors in livestock species. Vet Immunol Immunopathol. (2004) 102:351–62. 10.1016/j.vetimm.2004.06.008 15541789

[63] DiLillo DJ Palese P Wilson PC Ravetch JV. Broadly neutralizing anti-influenza antibodies require Fc receptor engagement for *in vivo* protection. J Clin Invest. (2016) 126:605–10. 10.1172/JCI84428 26731473 PMC4731186

[64] Henry Dunand CJ Leon PE Huang M Choi A Chromikova V Ho IY. Both neutralizing and non-neutralizing human H7N9 influenza vaccine-induced monoclonal antibodies confer protection. Cell Host Microbe. (2016) 19:800–13. 10.1016/j.chom.2016.05.014 27281570 PMC4901526

[65] Park SJ Si YJ Kim J Song MS Kim SM Kim EH. Cross-protective efficacies of highly-pathogenic avian influenza H5N1 vaccines against a recent H5N8 virus. Virology. (2016) 498:36–43. 10.1016/j.virol.2016.08.010 27543757

[66] Tan GS Leon PE Albrecht RA Margine I Hirsh A Bahl J. Broadly-reactive neutralizing and non-neutralizing antibodies directed against the H7 influenza virus hemagglutinin reveal divergent mechanisms of protection. PLoS Pathog. (2016) 12:e1005578. 10.1371/journal.ppat.1005578 27081859 PMC4833315

[67] Jacobsen H Rajendran M Choi A Sjursen H Brokstad KA Cox RJ. Influenza virus hemagglutinin stalk-specific antibodies in human serum are a surrogate marker for *in vivo* protection in a serum transfer mouse challenge model. MBio. (2017) 8:e01463–17. 10.1128/mBio.01463-17 28928215 PMC5605943

[68] Ye ZW Yuan S Poon KM Wen L Yang D Sun Z. Antibody-dependent cell-mediated cytotoxicity epitopes on the hemagglutinin head region of pandemic H1N1 influenza virus play detrimental roles in H1N1-infected mice. Front Immunol. (2017) 8:317. 10.3389/fimmu.2017.00317 28377769 PMC5359280

[69] Wang J Liu M Ding N Li Y Shao J Zhu M. Vaccine based on antibody-dependent cell-mediated cytotoxicity epitope on the H1N1 influenza virus increases mortality in vaccinated mice. Biochem Biophys Res Commun. (2018) 503:1874–9. 10.1016/j.bbrc.2018.07.129 30064910

[70] Shao W Li X Goraya MU Wang S Chen JL. Evolution of influenza A virus by mutation and Re-assortment. Int J Mol Sci. (2017) 18:1650. 10.3390/ijms18081650 28783091 PMC5578040

[71] Rajao DS Anderson TK Gauger PC Vincent AL. Pathogenesis and vaccination of influenza A virus in swine. Curr Top Microbiol Immunol. (2014) 385:307–26. 10.1007/82_2014_391 25033752

[72] Kitikoon P Vincent AL Janke BH Erickson B Strait EL Yu S. Swine influenza matrix 2 (M2) protein contributes to protection against infection with different H1 swine influenza virus (SIV) isolates. Vaccine. (2009) 28:523–31. 10.1016/j.vaccine.2009.09.130 19837089

[73] Khurana S Loving CL Manischewitz J King LR Gauger PC Henningson J. Vaccine-induced anti-HA2 antibodies promote virus fusion and enhance influenza virus respiratory disease. Sci Transl Med. (2013) 5:200ra114. 10.1126/scitranslmed.3006366 23986398

[74] Gauger PC Vincent AL Loving CL Lager KM Janke BH Kehrli ME Jr. Enhanced pneumonia and disease in pigs vaccinated with an inactivated human-like (delta-cluster) H1N2 vaccine and challenged with pandemic 2009 H1N1 influenza virus. Vaccine. (2011) 29:2712–9. 10.1016/j.vaccine.2011.01.082 21310191

[75] Crowe JE Jr. Universal flu vaccines: primum non nocere. Sci Transl Med. (2013) 5:200fs234. 10.1126/scitranslmed.3007118 23986396

[76] Morgan SB Holzer B Hemmink JD Salguero FJ Schwartz JC Agatic G. Therapeutic Administration of broadly neutralizing FI6 antibody reveals lack of interaction between human IgG1 and Pig Fc receptors. Front Immunol. (2018) 9:865. 10.3389/fimmu.2018.00865 29740451 PMC5928291

[77] Nachbagauer R Miller MS Hai R Ryder AB Rose JK Palese P. Hemagglutinin stalk immunity reduces influenza virus replication and transmission in ferrets. J Virol. (2015) 90:3268–73. 10.1128/JVI.02481-15 26719251 PMC4810634

[78] Yassine HM Boyington JC McTamney PM Wei CJ Kanekiyo M Kong WP. Hemagglutinin-stem nanoparticles generate heterosubtypic influenza protection. Nat Med. (2015) 21:1065–70. 10.1038/nm.3927 26301691

[79] Shen C Chen J Li R Zhang M Wang G Stegalkina S. A multimechanistic antibody targeting the receptor binding site potently cross-protects against influenza B viruses. Sci Transl Med. (2017) 9:eaam5752. 10.1126/scitranslmed.aam5752 29046433

[80] Bouvier NM Lowen AC. Animal models for influenza virus pathogenesis and transmission. Viruses. (2010) 2:1530–63. 10.3390/v20801530 21442033 PMC3063653

[81] Jegaskanda S Weinfurter JT Friedrich TC Kent SJ. Antibody-dependent cellular cytotoxicity is associated with control of pandemic H1N1 influenza virus infection of macaques. J Virol. (2013) 87:5512–22. 10.1128/JVI.03030-12 23468501 PMC3648138

[82] Florek NW Kamlangdee A Mutschler JP Kingstad-Bakke B Schultz-Darken N Broman KW. A modified vaccinia Ankara vaccine vector expressing a mosaic H5 hemagglutinin reduces viral shedding in rhesus macaques. PLoS ONE. (2017) 12:e0181738. 10.1371/journal.pone.0181738 28771513 PMC5542451

[83] Florek NW Weinfurter JT Jegaskanda S Brewoo JN Powell TD Young GR. Modified vaccinia virus Ankara encoding influenza virus hemagglutinin induces heterosubtypic immunity in macaques. J Virol. (2014) 88:13418–28. 10.1128/JVI.01219-14 25210172 PMC4249095

[84] Song A Myojo K Laudenslager J Harada D Miura T Suzuki K. Evaluation of a fully human monoclonal antibody against multiple influenza A viral strains in mice and a pandemic H1N1 strain in nonhuman primates. Antiviral Res. (2014) 111:60–8. 10.1016/j.antiviral.2014.08.016 25218949

[85] Cocklin SL Schmitz JE. The role of Fc receptors in HIV infection and vaccine efficacy. Curr Opin HIV AIDS. (2014) 9:257–62. 10.1097/COH.0000000000000051 24670320 PMC4120665

[86] Terajima M Co MD Cruz J Ennis FA. High antibody-dependent cellular cytotoxicity antibody titers to H5N1 and H7N9 avian influenza A viruses in healthy US adults and older children. J Infect Dis. (2015) 212:1052–60. 10.1093/infdis/jiv181 25795791 PMC4668882

[87] Jegaskanda S Vandenberg K Laurie KL Loh L Kramski M Winnall WR. Cross-reactive influenza-specific antibody-dependent cellular cytotoxicity in intravenous immunoglobulin as a potential therapeutic against emerging influenza viruses. J Infect Dis. (2014) 210:1811–22. 10.1093/infdis/jiu334 24916185

[88] Jegaskanda S Laurie KL Amarasena TH Winnall WR Kramski M De Rose R. Age-associated cross-reactive antibody-dependent cellular cytotoxicity toward 2009 pandemic influenza A virus subtype H1N1. J Infect Dis. (2013) 208:1051–61. 10.1093/infdis/jit294 23812238

[89] Co MD Terajima M Thomas SJ Jarman RG Rungrojcharoenkit K Fernandez S. Relationship of preexisting influenza hemagglutination inhibition, complement-dependent lytic, and antibody-dependent cellular cytotoxicity antibodies to the development of clinical illness in a prospective study of A(H1N1)pdm09 Influenza in children. Viral Immunol. (2014) 27:375–82. 10.1089/vim.2014.0061 25141276 PMC4183906

[90] Mesman AW Westerhuis BM Ten Hulscher HI Jacobi RH de Bruin E van Beek J. Influenza virus A(H1N1)2009 antibody-dependent cellular cytotoxicity in young children prior to the H1N1 pandemic. J Gen Virol. (2016) 97:2157–65. 10.1099/jgv.0.000552 27412007

[91] Vanderven HA Liu L Ana-Sosa-Batiz F Nguyen TH Wan Y Wines B. Fc functional antibodies in humans with severe H7N9 and seasonal influenza. JCI Insight. (2017) 2:e92750. 10.1172/jci.insight.92750 28679958 PMC5499372

[92] Vanderven HA Jegaskanda S Wines BD Hogarth PM Carmuglia S Rockman S. Antibody-dependent cellular cytotoxicity responses to seasonal influenza vaccination in older adults. J Infect Dis. (2017) 217:12–23. 10.1093/infdis/jix554 29106590

[93] Stadlbauer D Rajabhathor A Amanat F Kaplan D Masud A Treanor JJ. Vaccination with a recombinant H7 hemagglutinin-based influenza virus vaccine induces broadly reactive antibodies in humans. mSphere. (2017) 2:e00502–17. 10.1128/mSphere.00502-17 29242836 PMC5729220

[94] de Vries RD Altenburg AF Nieuwkoop NJ de Bruin E van Trierum SE Pronk MR. Induction of cross-clade antibody and T-cell responses by a modified vaccinia virus ankara-based influenza A(H5N1) vaccine in a randomized phase 1/2a clinical trial. J Infect Dis. (2018) 218:614–23. 10.1093/infdis/jiy214 29912453 PMC6047453

[95] Bangaru S Zhang H Gilchuk IM Voss TG Irving RP Gilchuk P. A multifunctional human monoclonal neutralizing antibody that targets a unique conserved epitope on influenza HA. Nat Commun. (2018) 9:2669. 10.1038/s41467-018-04704-9 29991715 PMC6039445

[96] Chai N Swem LR Park S Nakamura G Chiang N Estevez A A broadly protective therapeutic antibody against influenza B virus with two mechanisms of action. Nat Commun. (2017) 8:14234 10.1038/ncomms14234 28102191 PMC5253702

[97] He W Tan GS Mullarkey CE Lee AJ Lam MM Krammer F. Epitope specificity plays a critical role in regulating antibody-dependent cell-mediated cytotoxicity against influenza A virus. Proc Natl Acad Sci USA. (2016) 113:11931–6. 10.1073/pnas.1609316113 27698132 PMC5081650

[98] Cox F Kwaks T Brandenburg B Koldijk MH Klaren V Smal B. HA antibody-mediated FcgammaRIIIa activity is both dependent on fcr engagement and interactions between HA and sialic acids. Front Immunol. (2016) 7:399. 10.3389/fimmu.2016.00399 27746785 PMC5040702

[99] Leon PE He W Mullarkey CE Bailey MJ Miller MS Krammer F. Optimal activation of Fc-mediated effector functions by influenza virus hemagglutinin antibodies requires two points of contact. Proc Natl Acad Sci USA. (2016) 113:E5944–51. 10.1073/pnas.1613225113 27647907 PMC5056099

[100] Yu F Song H Wu Y Chang SY Wang L Li W. A potent germline-like human monoclonal antibody targets a pH-sensitive epitope on H7N9 influenza hemagglutinin. Cell Host Microbe. (2017) 22:471–83.e475. 10.1016/j.chom.2017.08.011 28966056 PMC6290738

[101] de Vries RD Nieuwkoop NJ van der Klis FRM Koopmans MPG Krammer F Rimmelzwaan GF. Primary human influenza B virus infection induces cross-lineage hemagglutinin stalk-specific antibodies mediating antibody-dependent cellular cytoxicity. J Infect Dis. (2017) 217:3–11. 10.1093/infdis/jix546 29294018 PMC5853962

[102] Bangaru S Lang S Schotsaert M Vanderven HA Zhu X Kose N. A site of vulnerability on the influenza virus hemagglutinin head domain trimer interface. Cell. (2019) 177:1136–52.e1118. 10.1016/j.cell.2019.04.011 31100268 PMC6629437

[103] Bajic G Maron MJ Adachi Y Onodera T McCarthy KR McGee CE. Influenza antigen engineering focuses immune responses to a subdominant but broadly protective viral epitope. Cell Host Microbe. (2019) 25:P827-835.e6. 10.1016/j.chom.2019.04.003 31104946 PMC6748655

[104] Jegaskanda S Luke C Hickman HD Sangster MY Wieland-Alter WF McBride JM. Generation and protective ability of influenza virus-specific antibody-dependent cellular cytotoxicity in humans elicited by vaccination, natural infection, and experimental challenge. J Infect Dis. (2016) 214:945–52. 10.1093/infdis/jiw262 27354365 PMC4996149

[105] Bodewes R de Mutsert G van der Klis FR Ventresca M Wilks S Smith DJ. Prevalence of antibodies against seasonal influenza A and B viruses in children in Netherlands. Clin Vaccine Immunol. (2011) 18:469–76. 10.1128/CVI.00396-10 21209157 PMC3067385

